# Genetic and hosts characterization of hantaviruses in port areas in Hainan Province, P. R. China

**DOI:** 10.1371/journal.pone.0264859

**Published:** 2022-03-03

**Authors:** Qiu-wei Wang, Li Tao, Su-ying Lu, Chang-qiang Zhu, Le-le Ai, Yizhe Luo, Rong-bin Yu, Heng Lv, Yun Zhang, Chong-cai Wang, Wei-long Tan

**Affiliations:** 1 Department of Epidemiology, School of Public Health, Nanjing Medical University, Nanjing, China; 2 Huadong Research Institute for Medicine and Biotechnics, Nanjing, China; 3 School of Medicine & Holistic Integrative Medicine, Nanjing University of Chinese Medicine, Nanjing, China; 4 Hainan International Travel Healthcare Center, Haikou, China; University of Illinois College of Medicine, UNITED STATES

## Abstract

**Background:**

Hantaviruses (HVs) are major zoonotic pathogens in China that cause hemorrhagic fever with renal syndrome (HFRS) posing a major threat to people’s health. Hainan Province, an island located in Southeast China, is an ideal region for sea ports. The unique tropical monsoon climate in Hainan provides sufficient living conditions for rodents, which help spread HVs and other rodent-borne diseases. In the routine monitoring of hantavirus, there was no evidence that rodents in Hainan carried hantavirus. No patients infected with hantavirus were found in the past. However, the surveillance of HVs-carrying rodents covering the whole territory of Hainan has not stopped.

**Methodology/principal findings:**

For the monitoring of the prevalence of HVs in rodents and the search for theoretical reference for rodent control and HFRS prevention, a total of 60 rodents from 6 monitoring spots were trapped around main ports in Hainan between 2016 and 2019. HV positive samples were identified by a specific kit and sequenced. The data indicated that seven rodents (*Rattus norvegicus*) were positive for hantavirus with a positivity rate of 11.67%. Phylogenetic analysis suggested that the two complete sequence strains HN1 and HN4 in this research were highly similar to the sequence strains GZRn36 and GZRn148 isolated in Guangdong Province, and they located in the same phylogenetic tree branch which belongs to S2 subtype. Although the two partial sequences HT1 and HT2 isolated in Xisha Islands belong to S2 subtype according to the phylogenetic tree of L segment, they showed a great nucleotide difference with HN1 and HN4. We also found 13 amino acid variations compared with SEOV 80–39 and 6 amino acid mutations related to epitope, and the variations may reduce the effectiveness of the current HFRS vaccines used in humans.

**Conclusions/significance:**

The study indicated HVs carried by rodents found in Hainan Province may be transmitted from Guangdong Province through trading ports and carriage of goods by sea. So it is of great significance to strengthen the surveillance of rodents in port areas especially capture and eliminate rodents on ship. Timely elimination of host animals of hantavirus in port areas is necessary to prevent an outbreak of HVs disease.

## Introduction

Hantaviruses (genus *Orthohantavirus*, family *Hantaviridae*) are composed of more than 80 hantavirus-related viruses, which transmit among many mammalian hosts, including bats (*Chiroptera*), shrews, moles (*Insectivora*) and rodents (*Rodentia*) [1, 2]. Humans may develop hemorrhagic fever with renal syndrome (HFRS) and hantavirus pulmonary syndrome (HPS) because of exposure to hantavirus [3]. More than 50,000 cases were reported worldwide every year, with mortality rate of 12% (HFRS) and 40% (HPS) [4]. The pathogen related to HPS is classified as a category A pathogen by the National Institute of Allergy and Infectious Diseases (NIAID). At present, at least 15 species of hantaviruses have been found to be associated with HCPS. A total of 624 HPS cases have been reported in the United States in the past 20 years from 1994 to 2014, and the incidence rate is increasing [5, 6]. So far, at least seven different species of hantaviruses have been associated with HFRS [7]. HTNV and SEOV are the main species of hantavirus prevalent in China respectively carried by *A*. *agrarius* and *R*. *norvegicus*. In Asia, 40,000 to 60,000 hantavirus-related cases are reported annually, 99% of which are from China [8]. Among them, severe cases of HFRS are caused by HTNV and Amur/Soochong viruses, with a mortality rate of up to 15% [7], while SEOV can lead to a moderate form of the disease with a mortality rate of 1%-2% [9].

With the development of import and export trade all over the world, climate change and deforestation, rodents were passively transported to other places and their population were increasing. To a large extent, this phenomenon increased the probability of rodent-human contacts [10]. Hantavirus is a single-stranded negative-strand RNA virus, which varies greatly. And there was still a lack of effective vaccine at present [11]. It is expected that the number of HFRS cases will continue to increase for a period of time [12]. The passive migration of the hantavirus-related hosts will not only lead to new plague foci, but also cause the controlled foci to rage again, particularly the ports, coastal locations and logistics bases [13].

Located in the South China Sea, Hainan Province is adjacent to Guangdong Province to the north, demarcated by Qiongzhou Strait and is opposite to Guangxi Province and Vietnam to the west with Beibu Gulf in between. It faces Taiwan Province to the east and the Philippines, Brunei, and Malaysia to the south. Hainan is known for its sea ports, such as Haikou, Sanya, Basuo, Yangpu and other port areas [14]. It has a unique tropical monsoon climate, with an annual average temperature of 22–27°C, and an annual precipitation between 1000 mm and 2600 mm. The island provides an adequate living environment for rodents and good conditions for the spread and prevalence of hantavirus [15]. The traffic between Hainan and domestic and foreign destinations mainly depends on shipping, ferries, airports and so on. There were many hantavirus-related investigations in the history of Hainan, but it was only detected in *R*. *norvegicus* in 2000 [16]. The import of hantavirus-related hosts in the province is mainly associated with trade and tourism which depend on aircrafts and ships etc. So, it is of great significance to investigate the epidemic status of hantavirus in Hainan, especially in wharf and port areas. And it is of great importance to strengthen hantavirus surveillance and provide support for hantavirus prevention and control in this area.

## Methods

### Ethics statement

China Customs in Hainan approved the collection of rodent samples. The Huadong Research Institute for Medicine and Biotechniques approved the relative research of hantavirus.

### Study of rodent samples

**Fig 1:**
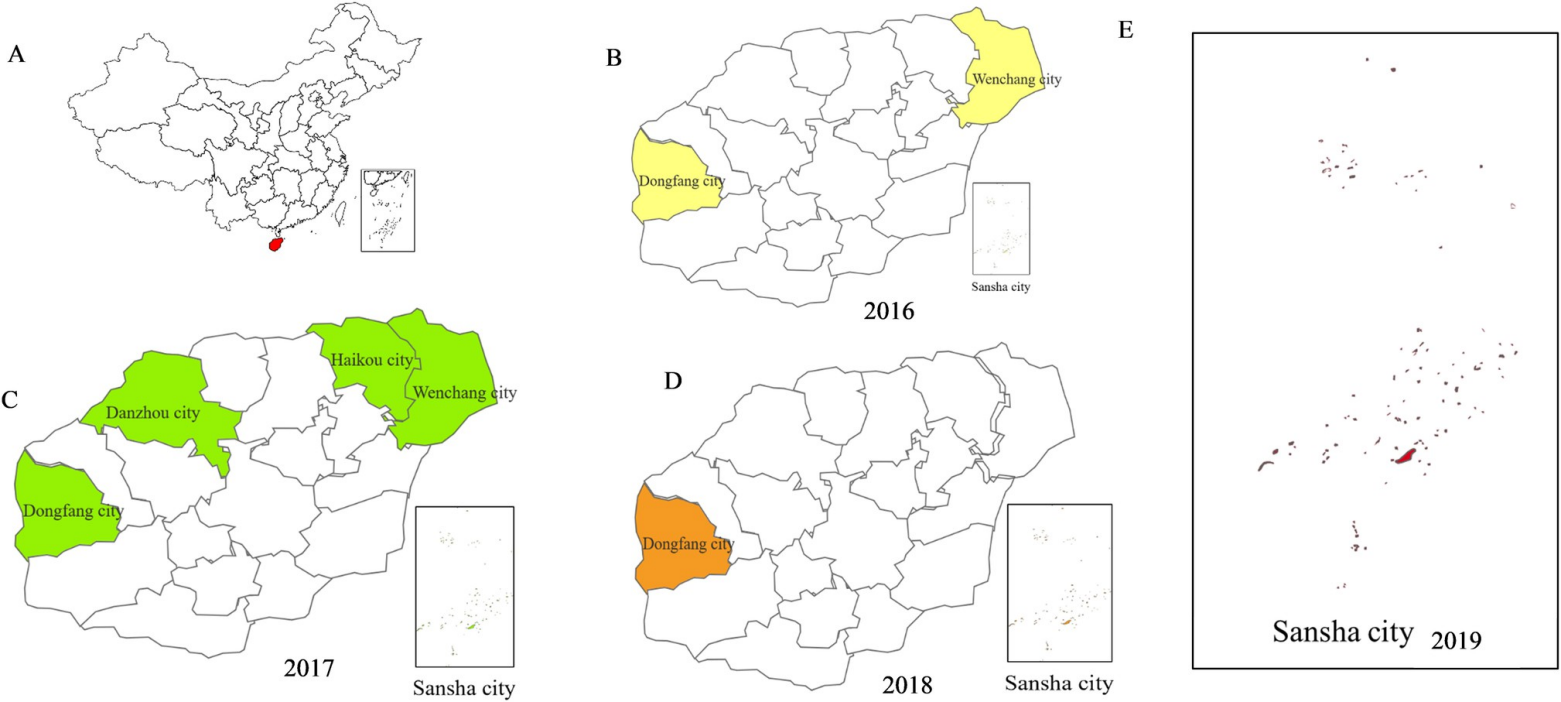
The sampling cities of rodents in Hainan Province from 2016 to 2019. A: The map of China. B: The sampling locations in 2016 including Dongfang and Wenchang. C: The sampling locations in 2017 including Dongfang, Danzhou, Haikou and Wenchang. D: The sampling locations in 2018 including Dongfang and Sansha. E: The sampling locations in 2019 including Sansha. Note: The map source referred to maps at the CIA which was similar but not identical to the original image and was therefore for illustrative purposes only.

**Fig 2:**
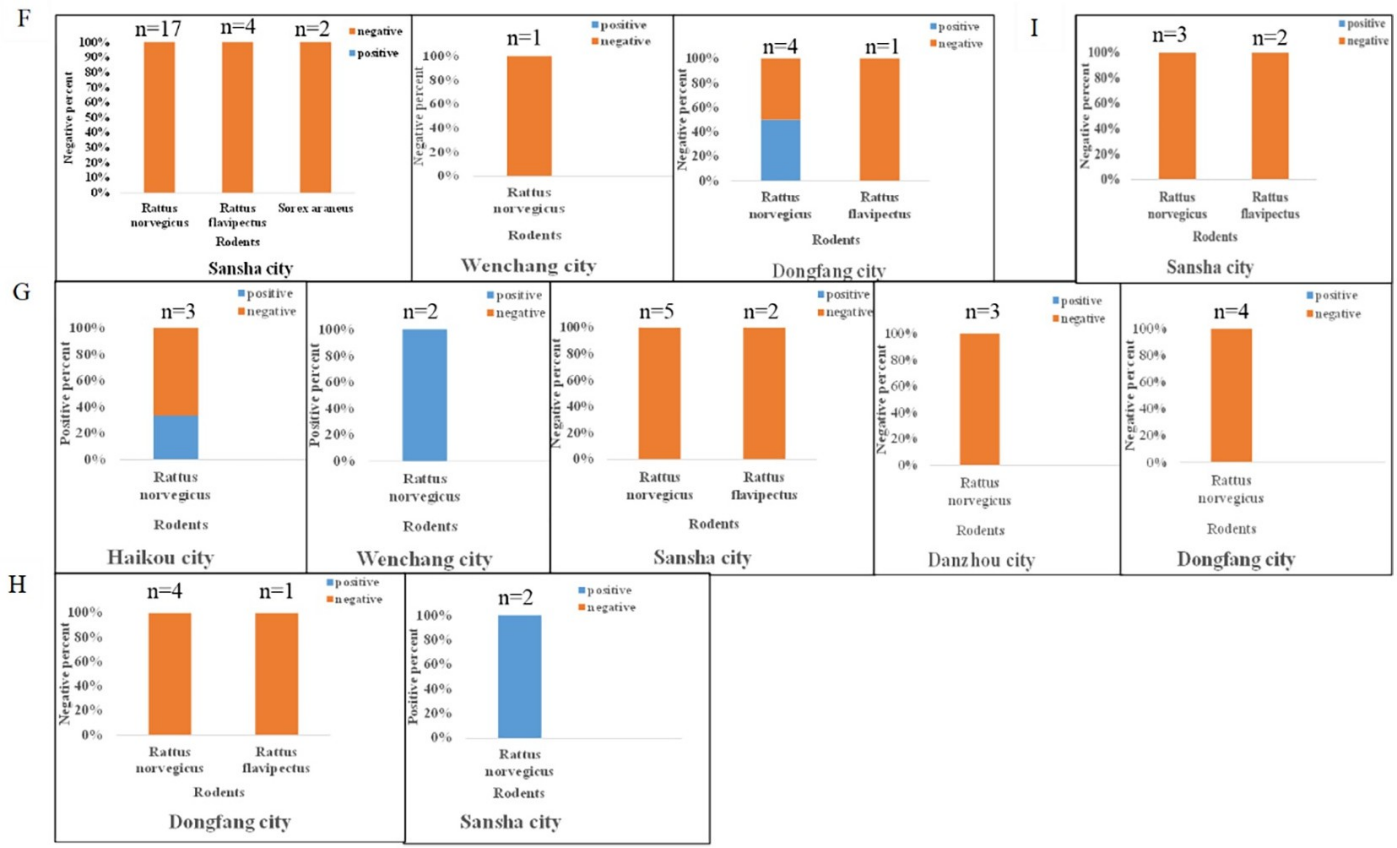
The positive rate in different cities during 2016–2019. F: The positive rate of hantavirus in Hainan Province in 2016. G: The positive rate of hantavirus in Hainan Province in 2017. H: The positive rate of hantavirus in Hainan Province in 2018. I: The positive rate of hantavirus in Hainan Province in 2019.

From 2016 to 2019, rodents were captured with traps, cages and sticky mouse boards amount to 500 which were generally set every five meters and baited with peanuts in 7 ports and their surrounding areas (including wharf, administrative areas, cafeterias and so on) in Hainan Province and were arranged in the evening and collected in the morning. All rodents captured were anesthetized and dissected. Muscle, lung and kidney tissues were collected and stored at -80°C. All rodents were collected in six areas, including Yongxing Island and Xisha Islands in Sansha City, Qingzha Port in Wenchang City, Basuo Port in Dongfang City, Haikou Port in Haikou City and Yangpu Port in Danzhou City (Figs 1 and 2). The rodent species were determined by morphological identification and cytochrome b gene.

### Identification of rodents

Firstly, rodent species were identified preliminarily on the basis of their appearance. 30 mg muscle tissues were grinded with 1ml PBS. Total DNA was extracted from rodent muscle tissues using the TaKaRa MiniBEST Universal Genomic DNA Extraction Kit. The primers L14724F and H15915R designed according to rodent’s cytochrome b gene were synthesized for PCR amplification [17]. The total volume of each PCR reaction was 50μl and contained 5μl of DNA sample, 2μl of the forward and reverse primers respectively and 41μl r-Taq DNA polymerase. The PCR amplification conditions included initial activation of the polymerase at 94°C for 3 min, 35 cycles at 94°C for 1 min, 50°C for 1 min, and 72°C for 1 min, followed by final extension at 72°C for 7 min. PCR products were gel-purified with a gel concentration of 1.3%. Positive PCR products were sequenced by Sangon Biotech (Shanghai) Co., Ltd..

### RNA extraction and hantavirus detection

All lung tissues were homogenized in the DMEM medium and filtered with 0.2μm filter (Millipore, Billerica, MA). Total RNA was extracted from rodent lung tissues using the QIAamp Viral RNA Mini Kit. The hantaviruses were detected by the Hantavirus Type I and II Universal Nucleic Acid Kit using fluorescence PCR method. The PCR reaction was run under the following conditions: 1 cycle, 10 min at 45°C and 15 min at 95°C; 40 cycles, each cycle consisting of 15 s at 95°C and 1 min at 60°C. Samples with a CT value ≤ 38 were considered positive for hantavirus. The positive samples of hantavirus were sent to Beijing Macro & Micro-test Bio-Tech Co., Ltd. for sequencing by Next generation sequencing(NGS).

### Genetic characterization and phylogenetic analysis

The genetic characteristics analysis and multiple sequence alignment of hantavirus were carried out using DNAstar software. The nucleotide and amino acid similarity and variation analysis were performed between the newly discovered hantavirus sequences in Hainan and other hantavirus sequences. The best substitution models, as selected by using MEGA-X, were estimated as the GTR + G for L, M and S-segment nucleotide sequences. Phylogenetic trees were constructed with the maximum-likelihood method based on the open reading frame (ORF) of L, M and S nucleotide segments, using MEGA-X. Node support was evaluated by bootstrap analysis of 1000 iterations. The clades were shown with bootstrap values ≥95%.

### Nucleotide sequence accession numbers

The S- and M- and partial L-segment sequences of hantaviruses described in this study have been deposited in GenBank with accession numbers MZ670777–MZ670782. Other previously published sequences used in the study were obtained from GenBank in S1 Table.

### Amino acid substitution analysis

According to previous studies, the M segment is the fastest mutated segment of hantavirus, and the mutation of this segment can cause changes in virus virulence, antigenicity, and cell fusion [18]. Amino acid variations between HN1 and HN4 and SEOV 80–39 and vaccine strains were studied using DNAMAN software.

## Results

### Hantavirus infection in rodents

A total of 60 rodents were captured from the ports in Hainan from 2016 to 2019 and there were 48 (80%) *R*. *norvegicus*, 10 (16.67%) *R*. *flavipectus*, 1 (1.67%) *Sorex araneus* and 1 (1.67%) *R*. *norvegicus*. Lung tissues were screened using fluorescence PCR to detect hantavirus RNA, and 7 (11.67%) *R*. *norvegicus* samples were identified positive. The numbers of trapped and hantavirus positive animals at each location are summarized in Table 1. The positive animals are mainly distributed in Xisha Islands in Sansha, Qingzha Port in Wenchang, Basuo Port in Dongfang, and Haikou Port in Haikou. In summary, hantavirus was detected in *R*. *norvegicus*, which was the predominant rodent species in the province.

**Table 1:** Detection of hantavirus in rodents in Hainan Province from 2016 to 2019.

Year	Place	*Apodemus agrarius*	*Rattus norvegicus*	*Mus musculus*	*Sorex araneus*	*Rattus flavipectus*	Total (positive/total)
		(positive/total)	(positive/total)	(positive/total)	(positive/total)	(positive/total)	
**2016**	Yongxing Island, Sansha City	-	0/17	-	0/2	0/4	0/23
	Qingzha Port, Wenchang City	-	0/1	-	-	-	0/1
	Basuo Port, Dongfang City	-	2/4	-	-	0/1	2/5
**2017**	Haikou Port, Haikou City	-	1/1	-	-	-	1/1
	Qingzha Port, Wenchang City	-	2/2	-	-	-	2/2
	Yongxing Island, Sansha City	-	0/5	-	-	0/2	0/7
	Meilan District, Haikou City	-	0/2	-	-	-	0/2
	Yangpu Port, Danzhou City	-	0/3	-	-	-	0/3
	Basuo Port, Dongfang City	-	0/4	-	-	-	0/4
**2018**	Basuo Port, Dongfang City	-	0/4	-	-	0/1	0/5
	Xisha Islands, Sansha City	-	2/2	-	-	-	2/2
**2019**	Yongxing Island, Sansha City	-	0/3	-	-	0/2	0/5

### Sequence analysis of hantavirus

Two complete sequences of hantavirus (HN1 and HN4) and two partial sequences of L segment (HT1 and HT2) were obtained from 7 positive samples. All sequences belong to SEOV hantavirus according to National Center Biotechnology Information (NCBI) BLAST. The L segments length of HN1 and HN4 strains are respectively 6530bp and 6512bp with ORF 37-6492bp encoding 2151 amino acids constituting RNA-dependent RNA polymerase. The M segments of HN1 and HN4 consist of 3645bp and 3546bp respectively and their ORF positions are 44-3445bp and 39-3374bp, which are composed of 1133 and 1111 amino acids that fall into the region of Glycoprotein. The length of S segments is 1769bp and 1725bp, and their ORF positions are 43-1332bp and 19-1308bp, encoding 429 amino acids forming nucleoprotein. The L segments of HT1 and HT2 are composed of 351bp and encode 116 amino acids.

### Genetic analyses of hantavirus sequences

The similarity between HN1 and HN4 strains isolated from Qingzha Port in Wenchang was higher, and the nucleotide and amino acid similarities of L segments were respectively 97.7% and 99.6%. The partial sequences HT1 and HT2 of the L segment isolated from Xisha Islands in Sansha were closely related with 98% nt similarity and 100% aa similarity. All sequences of L segment from the Hainan isolates were closely related with 94.6% to 96% nt similarity and 94.8% to 95.7% aa similarity. In addition, the nucleotide similarity between the L segment sequences of the four strains and hantavirus strains sequences isolated from Guangzhou and Shenzhen was 94% -99.2% (Table 2). The M segment analysis of HN1 and HN4 showed respectively 96.9% and 99.5% nucleotide and amino acid similarity. The two strains of M segment showed 97%- 99.3% nucleotide similarity with the HN71-L sequence found previously in Hainan. In addition, there was a high similarity between HN1 and HN4 in Hainan and GZRn76 and GZRn148 isolated from Guangdong province based on M segment, with nucleotide and amino acid similarity of 96.7%-98.2% and 99.4%-99.7%, respectively (Table 3). The nucleotide and amino acid similarity of the S segment of HN1 and HN4 were respectively 98.3% and 100%, which showed 97.5%-99.3% nt similarity and 99.7% aa similarity, respectively with GZRn76 and GZRn148 strains from Guangdong (Table 4).

**Table 2:** Analysis of the nucleotide and amino acid similarity of hantavirus L segment in Hainan Province and surrounding areas.

Virus strains	HN1	HN4	HT1	HT2	2013SZ32	GZRn76	GZRn148
**HN1**	***	99.6	94.8	95.7	100	99.8	99.6
**HN4**	97.7	***	94.8	95.7	100	99.8	99.6
**HT1**	96	95.1	***	100	94.7	94.8	94.8
**HT2**	94.9	94.6	98	***	95.6	95.7	95.7
**2013SZ32**	99.2	98.9	95.9	96.2	***	100	100
**GZRn76**	98	97.7	94.6	94	98.3	***	99.8
**GZRn148**	97.8	97.8	94.9	94.3	99.2	98.8	***

Note: The upper triangle region represents amino acid similarity and the lower triangle region indicates the nucleotide similarity.

**Table 3:** Analysis of the nucleotide and amino acid similarity of hantavirus M segment in Hainan Province and surrounding areas.

Virus strains	HN1	HN4	HN71	GZRn76	GZRn148	HB55
**HN1**	***	99.5	100	99.6	99.4	98.9
**HN4**	96.9	***	100	99.7	99.7	99
**HN71**	97	99.3	***	100	100	100
**GZRn76**	98.2	96.7	97.3	***	99.6	99.1
**GZRn148**	97.3	97.6	98.3	98.1	***	98.9
**HB55**	96.1	96.1	96	95.9	96	***

Note: The upper triangle region represents amino acid similarity and the lower triangle region indicates the nucleotide similarity.

**Table 4:** Analysis of the nucleotide and amino acid similarity of hantavirus S segment in Hainan Province and surrounding areas.

Virus strains	HN1	HN4	GZRn76	GZRn148
**HN1**	***	100	99.7	99.7
**HN4**	98.3	***	99.7	99.7
**GZRn76**	98.2	98.6	***	99.3
**GZRn148**	97.5	99.3	98.1	***

Note: The upper triangle region represents amino acid similarity and the lower triangle region indicates the nucleotide similarity.

### Genetic analysis of hantavirus among different genotypes and subtypes

In a comparison of different subtypes of SEOV and other species of hantavirus, it was found that HN1 and HN4 strains sequences in Hainan showed the highest similarity with S2 subtypes such as GZRn76 and GZRn148. The two strains sequences were closely related to S1 subtypes such as L99 and K24-e7, S3 subtypes such as ZT10 and FJ372, and S4 subtypes such as SEOV80-39 and SR11 with 94.4%-97% nt similarity and 98.1% -100% aa similarity, respectively. The two strains sequences (HN1 and HN4) were quite different from Gou3, ZJ5 and other S5 subtypes, and the nucleotide and amino acid similarities were respectively 84%-89.2% and 96.8%-99.3%. The similarity between the two partial L segments HT1 and HT2 and other seoul virus subtypes (S1, S3 and S4 subtypes) was low, which showed 90%-93.7% nucleotide similarity and 94%-95.7% amino acid similarity. There was obvious difference between the four strains sequences found in Hainan and HTNV76-118 with 69.1%-74.8% nucleotide similarity And nucleotide and amino acid similarity was 58.8%-66.9% and 53.8% - 68.7% compared with PUUV (Table 5).

**Table 5:** Sequence analysis of Hantavirus in Hainan with other genotypes and subtypes.

Virus strains	HN1		HN4		HT1		HT2	
	nucleotide	amino acid	nucleotide	amino acid	nucleotide	amino acid	nucleotide	amino acid
L99	94.8–96	98.9–99.3	94.9–96.2	99.1–99.3	91.1	94	91.1	94.8
K24-e7	96	98.9–99.5	95.9–96.6	99–99.5	-	-	-	-
GZRn76	98–98.2	99.6–99.8	96.7–98.6	99.7–99.8	94.6	94.8	94	95.7
GZRn148	97.3–97.8	99.4–99.7	97.6–99.3	99.6–99.7	94.9	94.8	94.3	95.7
ZT10	94.8–96.1	98.1–99.3	95–96.5	98.1–99.5	91.7	94.8	91.7	95.7
FJ372	94.4–96.5	99.3–99.5	94.7–97	99.3–99.8	90	94	90	94.8
SEOV80-39	94.9–96.6	99–100	95–96.8	99.1–100	90	94.8	91.1	95.7
SR11	95.4–95.7	98.4–99.1	95.6–95.7	98.4–99.5	-	-	-	-
GOU3	84.7–88.8	97–99.3	84.5–89.1	97.4–99.3	-	-	-	-
ZJ5	84–89.2	96.8–99.3	84–89	97.5–99.3	-	-	-	-
IR461	94.5–95.7	98.2–99.3	94.4–96	98.7–99.3	93.7	94.8	93.1	95.7
HTNV76-118	70.5–74.8	77.1–85.4	71.1–74.6	77.9–85.3	69.1	76.7	70	77.4
PUUV	59.1–66.5	53.8–68.7	58.8–66.3	54.8–68.6	66.9	60.3	66.6	60.9

Note: HN1 and HN4 strains were isolated from Hainan island; HT1 and HT2 strains were isolated from Xisha island of Sansha City.

### Phylogenetic analysis and conjecture of epidemic focus

**Fig 3:**
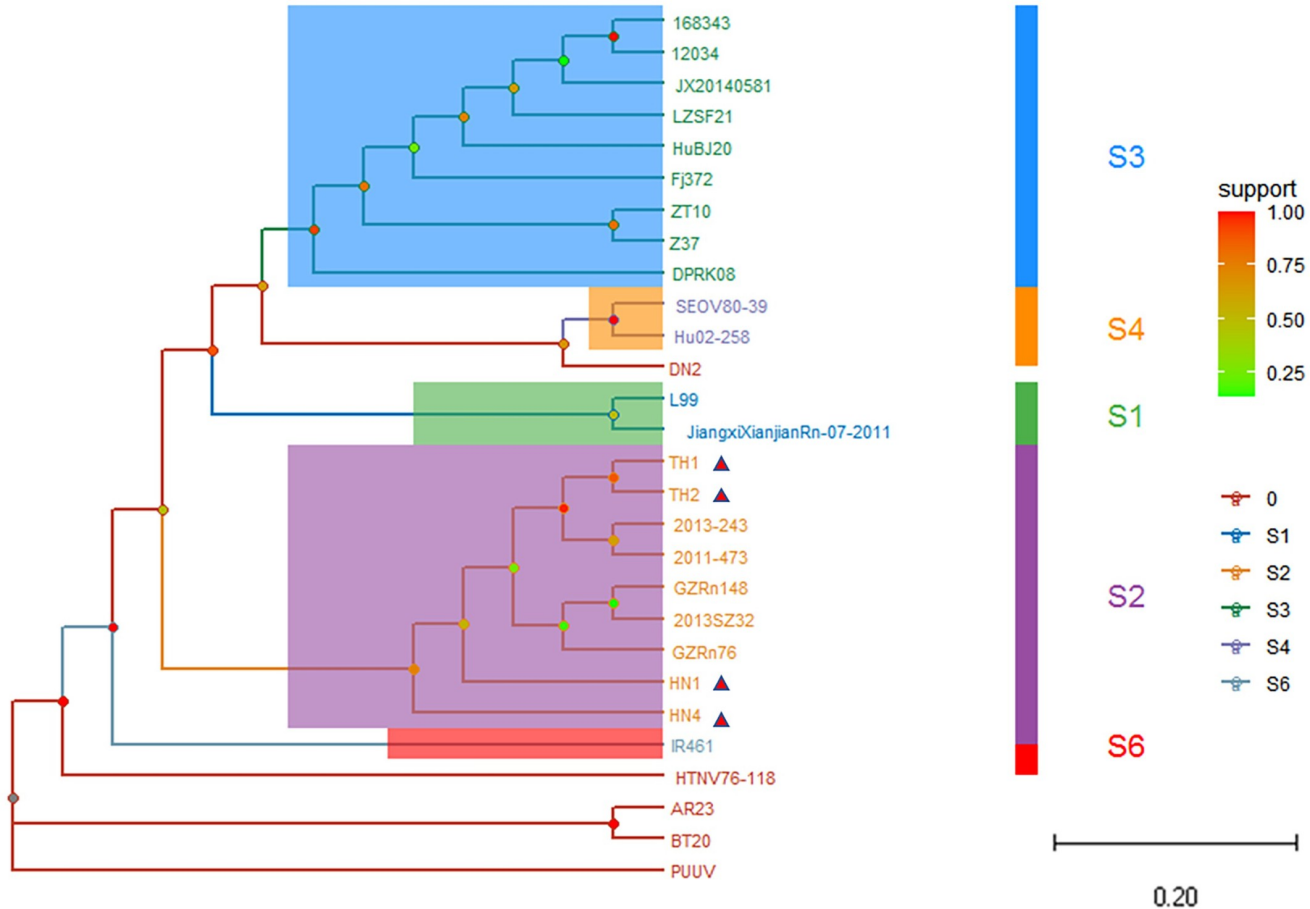
The phylogenetic tree based on ORF of L segment nucleotide sequences of hantavirus in Hainan and others. According to the phylogenetic analysis, the “L” sequences of the four hantavirus strains sequences found in Hainan Province belong to S2 subtype of SEOV. Note: The small red triangle represents the newly discovered hantavirus sequences in this study.

**Fig 4:**
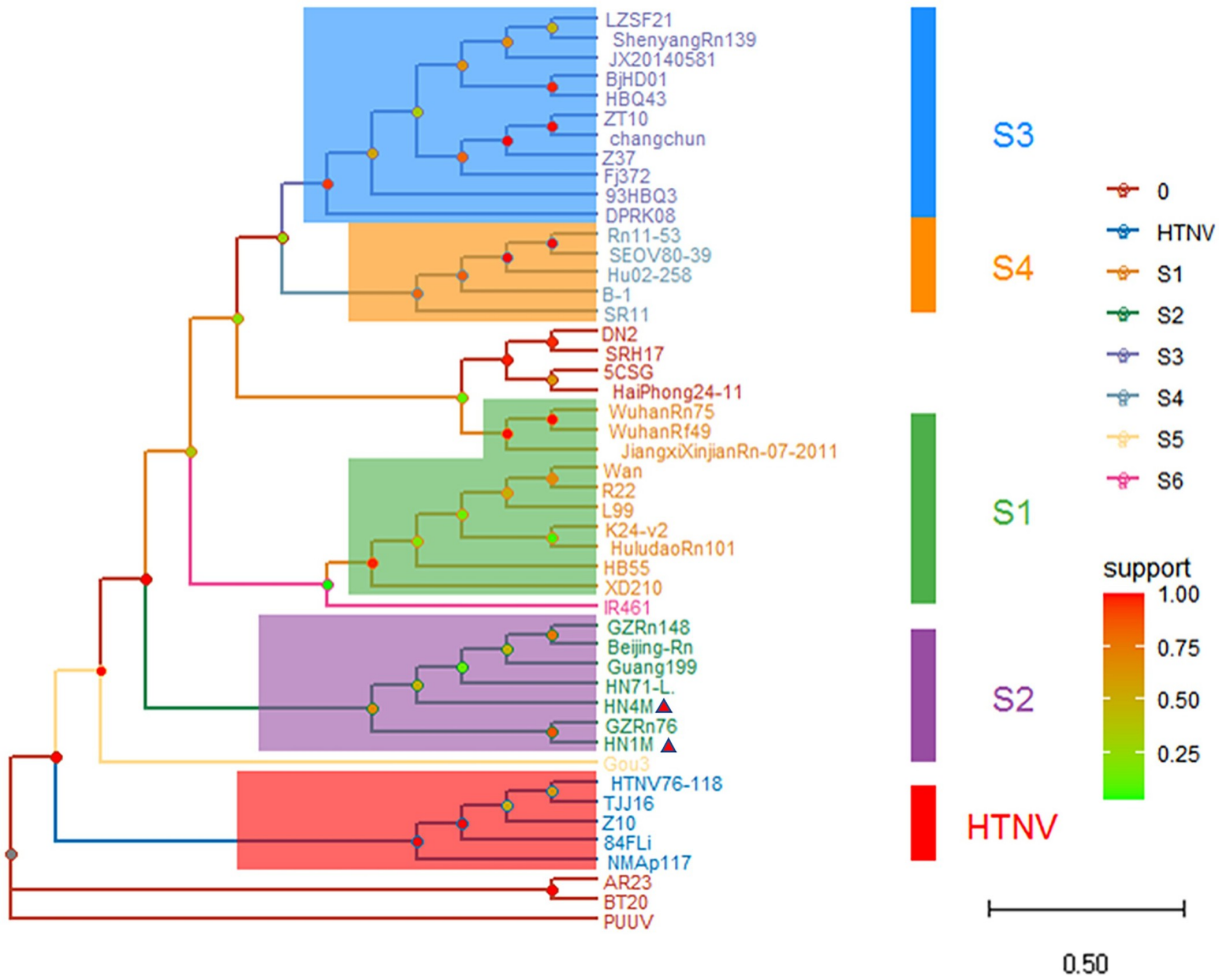
The phylogenetic tree based on ORF of M segment nucleotide sequences of hantavirus in Hainan and others. The ‘M’ sequences (named HN1M and HN4M) of the two hantavirus HN1 and HN4 show a close genetic distance with GZRn76, HN71-L, Guang199 and Beijing-Rn, which belong to S2 subtype. Note: The small red triangle represents the newly discovered hantavirus sequences in this study.

**Fig 5:**
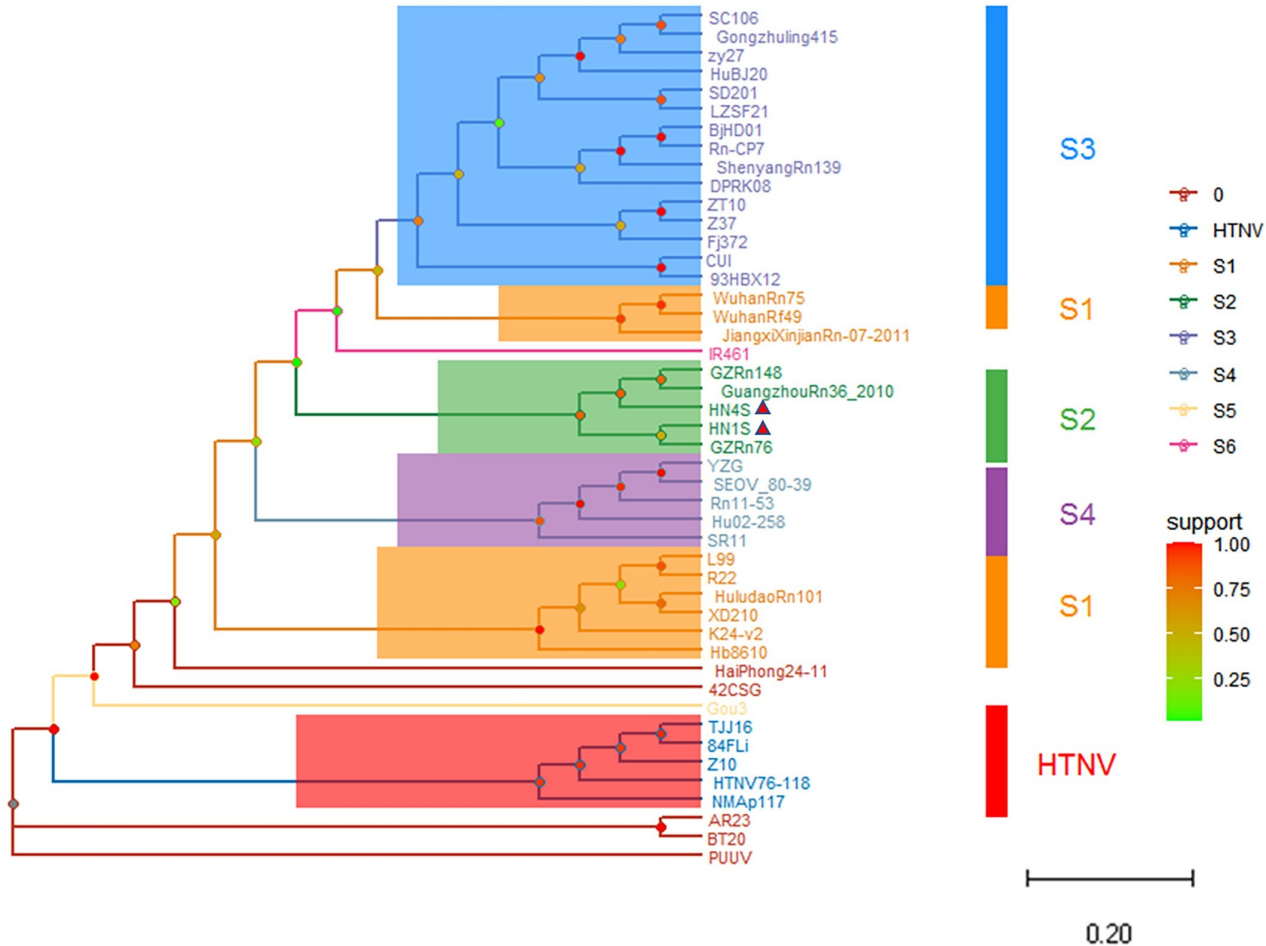
The phylogenetic tree based on ORF of S segment of hantavirus nucleotide sequences in Hainan and others. The HN1 and HN4 are located in the same branch of the phylogenetic tree with GZRn76, GZRn148 and GuangzhouRn36 isolated from Guangzhou. Note: The small red triangle represents the newly discovered hantavirus sequences in this study.

Based on the GTR+G evolution model, the phylogenetic trees of hantavirus newly discovered in Hainan and its neighboring provinces and different subtypes were constructed according to ORF of L, M and S segments. The topological of L segment phylogenetic tree shows that all sequence isolates of HN1, HN4, HT1, HT2 found in Hainan belong to S2 subtype, and there is a great genetic distance between the HN1 and HN4 and HT1 and HT2 (Fig 3). In the phylogenetic tree based on M-segment sequences, the HN1 and HN4 genome isolates fall into S2 subtype, which are in the same branch as GZRn76, GZRn148 and Guang199 hantavirus strains isolated from Guangdong (Fig 4). The topological structure of phylogenetic tree based on S segment is similar to that of the M segment, and the HN1 and HN4 are in the same branch of the phylogenetic tree as hantavirus of Guangdong Province (Fig 5). It is inferred that the newly discovered hantavirus may be imported into Hainan through trade and shipping through the South China Sea from Guangdong Province. The classification criteria of type and subtype were referred to previously published literature [3].

### Amino acid variation among hantaviruses

According to previous studies on the glycoprotein of hantavirus, it is found that SEOV has 8 mutation hotspots currently [18]. In this study, 13 mutant amino acids were found by comparing HN1 and HN4 with the international standard strain SEOV80-39 (Table 6). Eight of them are exactly the same as the currently discovered mutation hotspots.

**Table 6:** The amino acid variation analysis between HN1 and HN4 and SEOV 80–39.

HN1 (position/amino acid)	HN4 (position/amino acid)	SEOV 80–39 (position/amino acid)
32 (N)*	10 (N)*	32 (K)*
96 (V)*	74 (V)*	96 (L)*
97 (V)*	75 (V)*	97 (M)*
164 (I)*	142 (I)*	164 (V)*
229 (S)	207 (G)	229 (G)
327 (A)*	305 (A)*	327 (T)*
513 (M)	491 (I)	513 (I)
608 (I)	586 (V)	608 (I)
682 (K)*	660 (K)*	682 (R)*
1006 (K)*	984 (K)*	1006 (R)*
1051 (L)*	1029 (S)*	1051 (L)*
1085 (V)	1063 (I)	1085 (I)
1101 (S)	1089 (N)	1101 (N)

*: mutation hotspots

**Fig 6:**
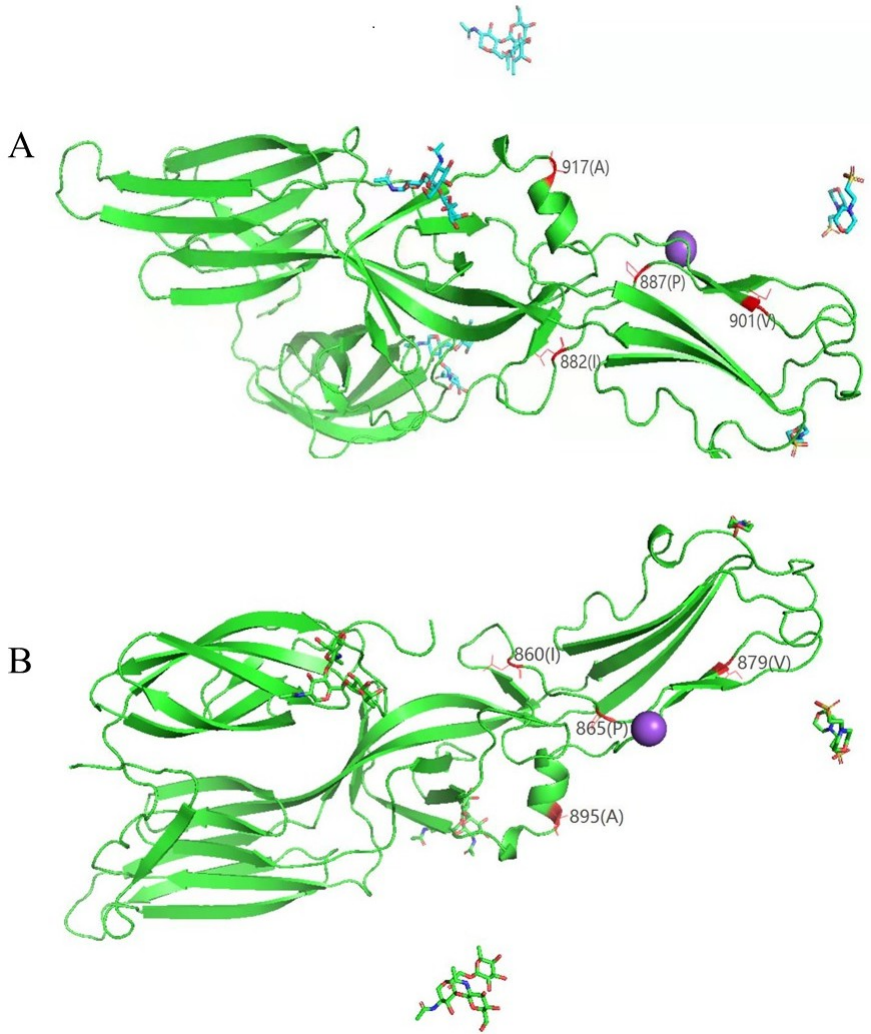
The tertiary structure of glycoprotein based on M segment. **A**: The amino acid variation analysis between HN1 and Z37 and L99 with the highest similarity to GC protein. **B:** The amino acid variation analysis between HN4 and Z37 and L99 with the highest similarity to GC protein.

We also compared HN1and HN4 with vaccine strains sequence (L99 and Z37), and found 6 amino acid variants related to antigenic epitope. There were 2 variations (P887A and A917E) between HN1 and HN4 and Z37 which are located in Gc glycoprotein. 4 variations including I79V and S88L in Gn glycoprotein and I882S and V901I in Gc glycoprotein were found between HN1 and HN4 and L99 (Table 7). We also used SWISS-MODEL and Pymol to build tertiary structure according to amino acid sequence of M segment. The tertiary structure of the protein with the highest similarity to GC is shown in Fig 6. The result showed the four antigenic epitopes of GC located on the surface of the tertiary structure of the protein. The variation may cause change in the antigenicity and virulence of strains and reduce the protective effect of existing vaccines.

**Table 7:** The amino acid variation analysis between HN1 and HN4 and Z37 and L99.

HN1 (position/amino acid)	HN4 (position/amino acid)	Z37 (position/amino acid)	L99 (position/amino acid)
79 (I)	57 (I)	-	79 (V)
88 (S)	66 (S)	-	88 (L)
882 (I)	860 (I)	-	882 (S)
887 (P)	865 (P)	887 (A)	-
901 (V)	879 (V)	-	901 (I)
917 (A)	895 (A)	917 (E)	-

## Discussions

In this paper, we described an investigative study on the presence of SEOV in seven port areas of Hainan, South China from 2016 to 2019. Hainan Island, with an area of 33,900 square kilometers, was formerly a region of Guangdong Province. It became a province, though the smallest one in China, in 1988. Ships, airplanes, and train ferries connect the island to the outside world and maintain its intensive contact with the mainland. In the past few years, outbreaks of hantavirus were rarely reported in Hainan, and host carrier rate was not yet clear [19]. In this study, 60 rodents were collected from Hainan and seven of them were hantavirus positive. The results revealed that the 7 positive rodents were *R*. *norvegicus* with 14.58% positive rate. According to infectious disease reports in China, the number of HFRS cases in Hainan has gradually increased in recent years (https://www.phsciencedata.cn/Share/). This could be that, with the rapid economic development and environmental changes, rodents were provided with good survival opportunities, such as suitable harbors, adequate food and water sources, and convenient transportation conditions [20]. In addition, in the urban environment, close contacts between rodents and people further increased the risk of disease transmission [21]. Although research showed that people in Hainan had a low infection rate at present, they lacked immunity to HFRS [22]. Once pathogens are imported, there is a great risk of epidemic and outbreak here.

In recent years, risk assessment and detection to medical vector and hantavirus in Hainan has been carried out, but hantavirus has not been detected in rodents [15, 19]. Because of the low infection rate of hantavirus, the population in Hainan is generally susceptible to the virus. Once the virus spreads to humans from rodents, it will seriously threaten the health of people. Therefore, the port monitoring of hantavirus in Hainan should be strengthened to prevent HFRS outbreaks. And rodents were captured regularly to detect the carrying status of Hantavirus and serological levels of people living in the area were tested regularly.

Genetic analyses of hantaviruses and their hosts will be helpful for understanding the changing epidemiology of HFRS. In this study, the majority of collected rodents in Hainan were *R*. *norvegicus*, which is similar to previous survey results [23], and the corresponding SEOV was predominant hantavirus. Similar patterns had been observed in other regions of China in recent years. For example, SEOV were isolated from *R*. *norvegicus* and *R*. *flavipectus* in Zhejiang Province [24]. Therefore, understanding the dominant rodent populations and the corresponding hantavirus genotypes in different regions plays an important role in controlling the spread of the disease.

According to the results of genetic characterization and phylogenetic analysis, it was found that the HN1 and HN4 of hantavirus in Hainan were similar to those isolated from Guangzhou and Shenzhen and belonged to the S2 subtype. Previous studies showed that hantavirus strains in Guangdong Province were mainly SEOV, and host animals are mainly *R*. *norvegicus*. So it might be possible that host animals carrying the seoul virus, especially brown rats, can migrate to Hainan by means of transportation from Guangdong Province [25]. In addition, the similarity analysis of sequences revealed that HT1 and HT2 isolated from Xisha Islands of Sansha were quite different from previous virus strains such as GZRn76 and GZRn148 with 94%-95% nucleotide similarity and HN1 and HN4 isolated from Qingzha Port of Wenchang. So it was speculated that the virus may have a greater variation. But due to various reasons, we only obtained partial sequences of HT1 and HT2, so we will further optimize and conduct in-depth research. In recent years, studies have shown that meteorological, geographical, production and living factors, etc. were of great significance to the mutation of hantavirus [26].Changes of climatic factors such as temperature, humidity, etc. and use of pesticides have caused influence on hantavirus spread [27]. In this study, 13 amino acid variations including 8 mutation hotspots were found between HN1 and HN4 and SEOV 80–39. The eight mutations are common in SEOV, and the mutation frequency exceeds 40%, which is common among hantaviruses. Besides, we also found 6 variations of glycoprotein related to epitope, which may cause antigenicity change [18]. The tertiary structure of glycoprotein based on HN1 and HN4 M segment was constructed. Four mutation sites related to antigenicity were located on the surface of the tertiary structure of the Gc protein. The variation may be related to antigen recognition, delivery and induction of immune response.

In addition, studies have shown that the high similarity between HN1 and HN4 and other subtypes of SEOV indirectly reflected that frequent communication among regions has promoted the spread of SEOV [28]. All data indicated that convenient transportation (especially water transportation) of hantavirus-carrying hosts and the abundance of such hosts may accelerate the migration of *R*. *norvegicus*, thereby accelerating the spread of SEOV in Hainan and other parts of China and even abroad. These studies supported the recent hypothesis that the current global distribution of SEOV was caused by the migration of Norwegian rats [29].

Hainan, the second largest island of China, is an ideal place for sea ports. The tropical and monsoon climate here is very suitable for rodent reproduction, which in turn facilitates the spread of hantavirus. Although not found in Hainan ports in previous studies, hantavirus-positive rodents existed in neighboring regions such as Guangzhou and Shenzhen [19, 30]. The findings in this study sounded an alert. We will continue to follow-up with the research and be prepared for human infection. The following study will establish a complete model for the formation of a new foci of hantavirus and research on prevention and control measures. Admittedly, the small sample size was one of the limitations of this paper.

In recent years, the infection of HVs showed an increasing trend, and the extent and scale of hantavirus outbreaks were increasing. On the one hand, hosts are rampant in some areas and environmental changes may affect geographical distribution, quantity and dynamics of rodent species due to climate change, economic development, mismanagement and other reasons, since the vaccine of HVs has not yet been widely applied [27]. On the other hand, with economic globalization and frequent trade activities, the import of hantavirus-carrying hosts made foci of imported cases appear everywhere [31, 32]. The trade between the three major ports of Hainan and other regions may provide suitable conditions for the spread and proliferation of SEOV. So the risk of hantavirus import in Hainan is very high, especially for port workers such as construction and sanitation workers, who may have occupational exposures. Standard preventive measures include rodent control, reducing rodent shelters and food sources near human habitation, eliminating rodents in homes, and avoiding contact with potentially contaminated areas. Above all, vaccines were the only effective way to reduce the risk of hantavirus. Therefore, for the sake of preventing the outbreak of hantavirus-related diseases, it is of great significance to strengthen the monitoring of hantavirus and its hosts in ports and surrounding areas in Hainan. We must understand the genetic characteristics of hantavirus and prepare ourselves for vaccine research and development. Immediate and strict measures of rodent control shall be taken in Hainan especially in its port areas before it is too late.

## Conclusion

In summary, the rate of hantavirus infection was gradually increasing in Hainan province. The SEOV virus founded in Hainan suggested that it can be transmitted from the surrounding areas. Besides, the variation of nucleotides and amino acids may render vaccines ineffective. Therefore, it is of great significance to explore the epidemic trend and variation of Hantavirus infection to prevent its epidemic.

## Supporting information

S1 Table The genbank number of sequences referred in the study. It includes all sequences used to construct phylogenetic trees.
(XLSX)
